# Soft Tissue Volume Augmentation at Single Implant Sites Applying Collagen Matrices or Connective Tissue Grafts: 10‐Year Follow‐Up of a Randomized Controlled Trial

**DOI:** 10.1111/jerd.70194

**Published:** 2026-05-19

**Authors:** Franz J. Strauss, Margherita G. Liguori, Thomas J. W. Gasser, Nadja Naenni, Ronald E. Jung, Daniel S. Thoma

**Affiliations:** ^1^ Discipline of Periodontics The University of Sydney, School of Dentistry Sydney New South Wales Australia; ^2^ Universidad Autonoma de Chile Santiago Chile; ^3^ Clinic of Reconstructive Dentistry University of Zurich Zurich Switzerland; ^4^ Private Practice Basel Switzerland; ^5^ Department of Periodontology, Research Institute for Periodontal Regeneration Yonsei University College of Dentistry Seoul South Korea

**Keywords:** aesthetics, collagen matrix, connective tissue, dental implants, patient‐reported outcome measures, peri‐implant mucosa, soft‐tissue augmentation

## Abstract

**Aim:**

To compare up to 10 years clinical, profilometric and patient‐reported outcomes of implant sites previously augmented using a volume‐stable collagen matrix (VCMX) or connective tissue graft (SCTG) in the aesthetic zone.

**Methods:**

The original non‐inferiority randomized controlled trial (RCT) enrolled 20 patients who received soft tissue volume augmentation with VCMX or SCTG at single implant sites. Clinical assessments and standardized measurements were performed at baseline after crown insertion and at 6 months, 1, 3, 5, 7.5, and 10 years. The primary outcome was mucosal thickness. Secondary outcomes included marginal bone levels (MBL), probing depth (PD), bleeding on probing (BOP), plaque control record, Pink Aesthetic Score (PES), OHIP‐14 and buccal profilometric changes. Group comparisons were performed using mixed‐effects and generalized estimating equation (GEE) models, which account for within‐patient correlations due to repeated measurements and allow inclusion of all available data without requiring imputation for missing observations.

**Results:**

Of the 20 originally enrolled patients, 10 (5 in the SCTG group and 5 in the VCMX group) were available for re‐examination at 10 years. The adjusted between‐group difference in mucosal thickness was −0.02 mm (95% CI −0.99 to 0.96). As the lower bound of the confidence interval remained above the prespecified non‐inferiority margin of −1 mm, non‐inferiority of VCMX was shown. Buccal contour changes were comparable during the early follow‐up, while a trend toward a greater long‐term contour decrease was observed in group VCMX (−0.31 mm [95% CI, −0.65 to 0.03]; *p* = 0.07). Mean PES values were 10.6 in the SCTG group and 9.6 in the VCMX group, with no significant between‐group differences (*p* = 0.45). Both groups revealed high levels of oral health–related quality of life, with low median OHIP‐14 scores (SCTG, 0.0; VCMX, 1.0; *p* = 0.26).

**Conclusion:**

These preliminary long‐term findings showed no clinically relevant differences between SCTG and VCMX in terms of clinical, profilometric and patient‐reported outcomes. While SCTG remains the reference standard, VCMX represents a less invasive alternative but with a slight tendency toward greater long‐term contour reduction.

**Clinical Significance:**

Volume‐stable collagen matrices serve as a viable alternative to autogenous connective tissue grafts for peri‐implant soft tissue volume augmentation, particularly in patients seeking a reduced morbidity, without compromising long‐term clinical or aesthetic outcomes.

**Trial Registration:** German Clinical Trials Register: DRKS00017484.

## 1 Introduction

Soft tissue augmentation is an important component of implant therapy, as increased peri‐implant mucosal thickness contributes to both long‐term tissue stability and improved aesthetic outcomes [1, 2, 3]. Depending on the clinical indication, these procedures may be performed prior to implant placement, at the time of surgery, or during the healing phase of the implant and even after the restorative phase [4].

From a clinical standpoint, increasing soft tissue volume provides several benefits. During early healing, it supports reconstruction of the natural ridge contour and facilitates the development of a stable peri‐implant soft tissue profile [5]. Over time, greater mucosal thickness has been associated with more favorable peri‐implant conditions, including reduced probing depths (PDs), lower plaque accumulation, decreased bleeding on probing (BOP), and more stable marginal bone levels [3, 6]. In addition, adequate tissue thickness is critical from an aesthetic perspective, particularly to prevent the visibility of underlying restorative components when the mucosa is thinner than 2 mm [7, 8]. Increased soft tissue volume has also been associated with improved long‐term stability of the mucosal margin [4, 9].

Autogenous subepithelial connective tissue grafts (SCTGs) are widely regarded as the reference standard for soft tissue augmentation because of their predictable biological performance and favorable aesthetic outcomes [10, 11, 12, 13]. However, their use is associated with increased morbidity related to graft harvesting, including postoperative pain and potential complications such as intraoperative bleeding, injury of the greater palatine artery, and tissue [14, 15, 16, 17]. Moreover, a recent clinical trial revealed that SCTG harvesting increases the physiological and psychological stress of the treating clinician [18]. In addition, anatomical factors may limit graft availability. In contemporary practice, where patient‐reported outcomes (PROs) are increasingly emphasized, these disadvantages are clinically relevant [19, 20, 21, 22].

For these reasons, soft tissue substitutes have been introduced as less invasive alternatives [14, 23, 24, 25]. Clinical studies have suggested that these materials achieve clinically meaningful increases in mucosal thickness, with outcomes that may approach those of connective tissue grafts while substantially reducing patient morbidity [14, 22, 26]. However, evidence regarding their long‐term performance remains limited. Most studies report outcomes up to 3 years [15, 23, 27], with only one study up to 7.5 years [28]. This represents an important gap, as previous studies have indicated that augmented tissues, particularly those obtained with substitutes, may undergo gradual dimensional changes over time [24, 27, 29]. Determining whether these materials can maintain peri‐implant tissue stability and satisfactory aesthetic outcomes over the long term is therefore essential for clinical decision‐making.

The present study reports the 10‐year outcomes of implant sites in the anterior region treated with either a soft tissue substitute or an autogenous connective tissue graft.

## 2 Materials and Methods

### 2.1 Study Design

This study represents a non‐interventional 10‐year follow‐up of a randomized controlled trial (RCT) [25] previously conducted at the Clinic of Reconstructive Dentistry, Center of Dental Medicine, University of Zurich, Switzerland. The original trial was designed in accordance with the CONSORT guidelines (Table S1) and the principles outlined in the Declaration of Helsinki. Ethical approval was obtained from the local ethics committee (Kantonale Ethikkommission Zürich; KEK‐ZH‐Nr 2011–0408) and the long‐term extension of the study received additional approval (KEK‐ZH‐Nr 2012–0226). The trial was registered in the (DRKS00017484) Clinical Trials Register. All participants provided written informed consent prior to enrolment and for participation in the extended follow‐up.

### 2.2 Eligibility Criteria

Participants were eligible for inclusion if they had been enrolled in the original RCT and had received a single fixed implant‐supported restoration in the anterior region. For the present follow‐up, patients were required to have completed the definitive prosthetic rehabilitation and to be available for long‐term reassessment. In addition, participants needed to demonstrate the ability to understand the study procedures and provide informed consent.

Patients were excluded if they developed systemic or local conditions that could interfere with soft tissue healing (e.g., uncontrolled diabetes), presented with peri‐implant infection unrelated to the initial augmentation procedure, underwent additional soft tissue augmentation at the study site, experienced trauma affecting the implant region, received orthodontic treatment in the same quadrant, or declined participation in the long‐term follow‐up.

### 2.3 Clinical Procedures

All 20 participants enrolled in the original RCT underwent a soft tissue augmentation procedure at a single‐tooth implant site that had healed under submerged conditions. After local anesthesia, incisions were made around the adjacent teeth to access the edentulous area. A horizontal incision was then extended across the crest, either straight or slightly shifted to the palatal/lingual side, connecting the mesial and distal line angles of the neighboring teeth. On the buccal aspect, a split‐thickness flap was carefully elevated to create a pouch that exceeded the dimensions required to accommodate the planned graft.

Intraoperatively, treatment allocation was disclosed using sealed envelopes. Patients were assigned to one of two interventions:

— VCMX: augmentation with a cross‐linked, volume‐stable collagen matrix (Geistlich Fibro‐Gide, Geistlich Pharma AG, Wolhusen, Switzerland).
— SCTG: augmentation with an autogenous SCTG harvested from the palate using a single‐incision technique.

In both groups, the graft material was shaped to fit the recipient site and inserted into the prepared pouch. In the VCMX group, the block (25 × 25 × 8 mm) was adapted and shaped to conform to the morphology of the recipient site. Stabilization was achieved with sutures, followed by tension‐free primary wound closure using a combination of mattress (Gore‐Tex 5‐0; W.L. Gore & Associates, Flagstaff, AZ, USA) and interrupted sutures.

Postoperative care included rinsing twice daily with 0.2% chlorhexidine solution (Hibitane; AstraZeneca), systemic anti‐inflammatory medication (Ponstan; Parke‐Davis), and systemic antibiotics (amoxicillin 2.25 g/day; Sandoz) for 7 days. To avoid trauma to the augmented area, temporary removable partial dentures were relined accordingly. Sutures were removed 7–10 days postoperatively. After a healing period of 3 months, implant uncovery was performed by abutment connection using a minimally invasive U‐shaped approach. Fixed provisional restorations were placed and maintained to allow soft‐tissue conditioning and contour maturation, followed by delivery of definitive screw‐retained single crowns. Patients were then scheduled for the baseline examination and enrolled in an individualized maintenance program throughout the entire observation period.

### 2.4 Follow‐Up Examinations

Baseline (BL) was defined as 2 weeks after crown delivery. Patients were evaluated at BL and at 6 months (FU‐6 M), 1 year (FU‐1), 3 years (FU‐3), 5 years (FU‐5), 7.5 years (FU‐7.5), and 10 years (FU‐10). All examinations were performed by a blinded examiner who was not involved in the original RCT and was unaware of the treatment allocation. Supportive maintenance visits were scheduled individually at intervals of 3 to 6 months.

### 2.5 Outcome Measures

#### 2.5.1 Primary Outcome Measure: Mucosal Thickness

The mucosal thickness was assessed using an endodontic file (K‐File 31/15) inserted 1 mm apical of the mucosal margin on the buccal side. Changes in mucosal thickness over time (BL to FU‐10) were considered as primary outcome.

#### 2.5.2 Secondary Outcome Measures

##### 2.5.2.1 Buccal Contour Changes

**FIGURE 1:**
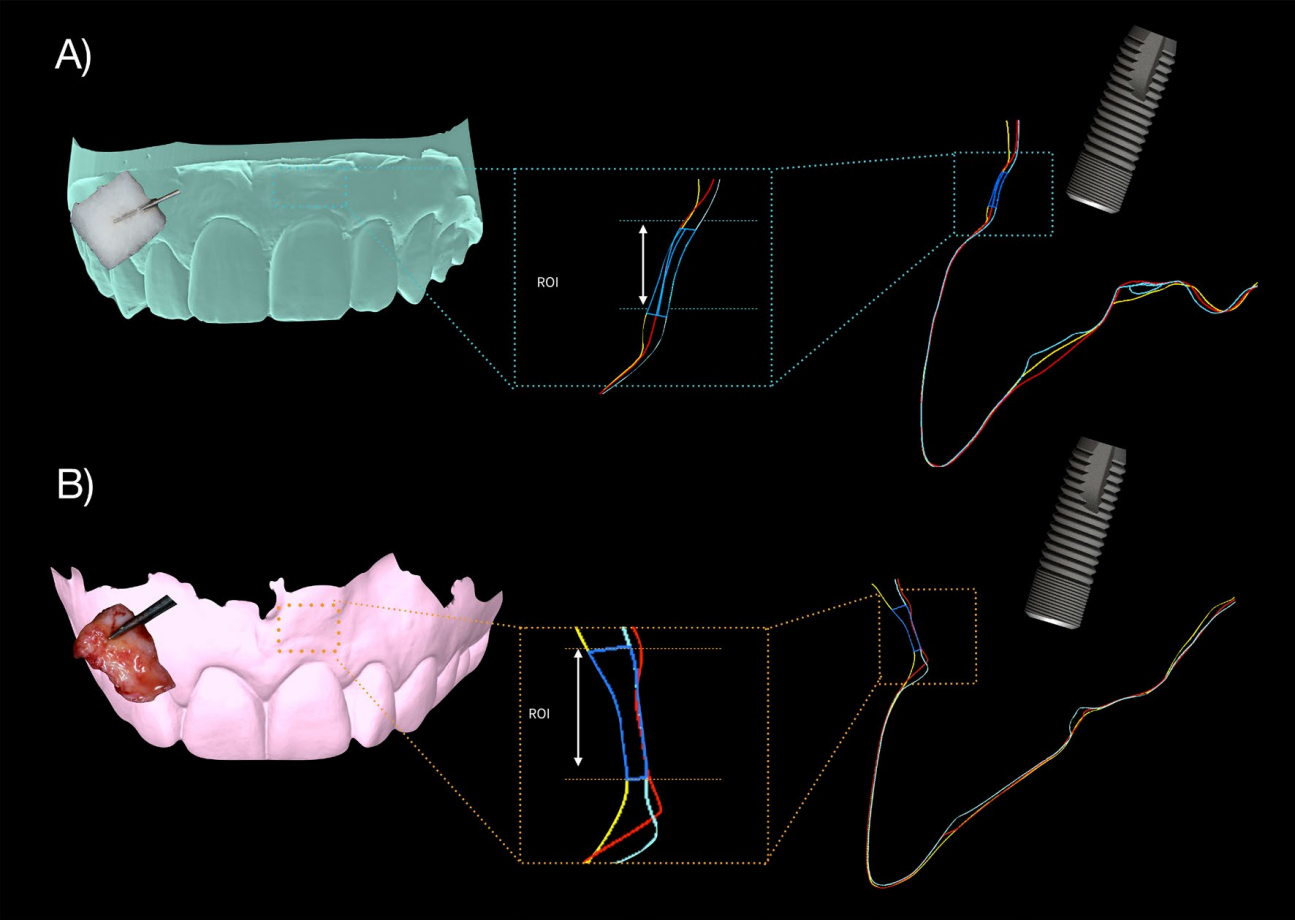
Digital workflow for the assessment of buccal contour changes based on the superimposition of serial intraoral scans in the VCMX (A) and SCTG (B) groups.

At each follow‐up visit, impressions of the implant site and the two adjacent teeth were taken using an A‐silicone material. Casts were poured and digitized with a laboratory scanner to obtain stereolithography (STL) files, which were then imported into a digital imaging software program (SMOP, Swissmeda, Zurich, Switzerland). For each case, a trapezoid‐shaped region of interest (ROI) was defined as follows: the coronal border was placed 1 mm apical to the mucosal margin, the apical border corresponded to the mucogingival junction and the mesial and distal borders were located 1 mm from the neighboring teeth. Due to interindividual anatomical differences, the absolute dimensions of the ROI varied between patients, but once established at the first assessment, the same ROI was applied consistently to all subsequent time points. STL datasets from baseline and the follow‐ups (6 months, 1, 3, 5, 7.5 and 10 years) were superimposed using a best‐fit alignment on the surfaces of the neighboring teeth. The software then calculated the mean surface distance between datasets within the ROI, expressed in millimeters (Figure 1).

##### 2.5.2.2 Clinical and Periodontal Measurements

PD, the plaque control record (PCR), and bleeding on probing (BOP) were assessed at six sites for all implants and the respective two neighboring teeth. The presence of peri‐implant health or disease was assessed according to the report of the 2017 World Workshop on the Classification of Periodontal and Peri‐Implant Diseases and Conditions [30].

##### 2.5.2.3 Diagnosis of Peri‐Implant Conditions

Peri‐implantitis was diagnosed according to the 2017 World Workshop [30]. Peri‐implant mucositis was defined according to the updated ID‐COSM consensus as bleeding (more than one spot on gentle probing) without bone loss beyond initial crestal bone remodeling [31].

##### 2.5.2.4 Radiologic Examination and Assessment of Bone Loss

Standardized periapical radiographs were obtained at all follow‐up visits using the long‐cone paralleling technique with digital sensor holders. Marginal bone levels were assessed by measuring the distance between the implant shoulder and the first bone‐to‐implant contact (fBIC) at the mesial and distal aspects of each implant. The mean of both sites was considered for analysis. Radiographic measurements were calibrated using known implant dimensions (inter‐thread pitch or implant length) to correct for potential radiographic distortion. All measurements were performed by a single examiner who was independent of the surgical and prosthetic procedures. To assess intra‐examiner reliability, measurements were repeated on two separate occasions at least 1 month apart. For the second assessment, radiographs from 10 patients were randomly selected using a computer‐generated sequence (www.randomizer.org).

Intra‐examiner reliability was assessed using the intraclass correlation coefficient (ICC) based on a two‐way mixed‐effects model with absolute agreement. The average ICC was > 0.9 for mesial sites and 0.9 for distal sites, indicating excellent agreement.

##### 2.5.2.5 Aesthetics

Peri‐implant soft‐tissue aesthetics were evaluated by a blinded, calibrated examiner using the Pink Aesthetic Score (PES) (Fürhauser et al. [32]).

##### 2.5.2.6 Patient‐Reported Outcomes

PROs were assessed at each follow‐up time point using a standardized and validated patient‐reported outcome measure (PROM) namely the validated German version of oral health impact profile‐G14 (OHIP‐G14).

### 2.6 Sample Size

The original sample size was calculated to assess the non‐inferiority of VCMX compared to SCTG for changes in mucosal thickness [25]. Assuming a non‐inferiority margin of 1 mm, an SD of 0.5 mm, and a 30% dropout rate, 10 patients per group were required to achieve 95% power at a one‐sided alpha of 0.025.

### 2.7 Statistical Analysis

Descriptive statistics (mean, SD, median, Q1, Q3) were calculated for all continuous variables. For the primary outcome (mucosal thickness), a non‐inferiority analysis was performed. Non‐inferiority of VCMX compared with SCTG was concluded if the lower bound of the two‐sided 95% confidence interval (CI) for the difference in mean mucosal thickness (VCMX − SCTG) was greater than −1 mm. The non‐inferiority test was restricted to the primary outcome; all secondary outcomes were analyzed using superiority testing. Group comparisons over time were performed using linear mixed‐effects models to account for repeated measurements within patients. The models included treatment group, time, and the group × time interaction as fixed effects, with patients included as random effects. Missing data were handled within the mixed‐effects modeling framework, which allows inclusion of all available observations without the need for imputation, assuming data were missing at random. Model assumptions were evaluated using residual diagnostics (*Q*–*Q* plots and histograms). When assumptions were not met, generalized estimating equations (GEEs) were applied. All analyses were performed using Stata version 19.5 (StataCorp, College Station, TX, USA).

## 3 Results

### 3.1 Patients

**FIGURE 2:**
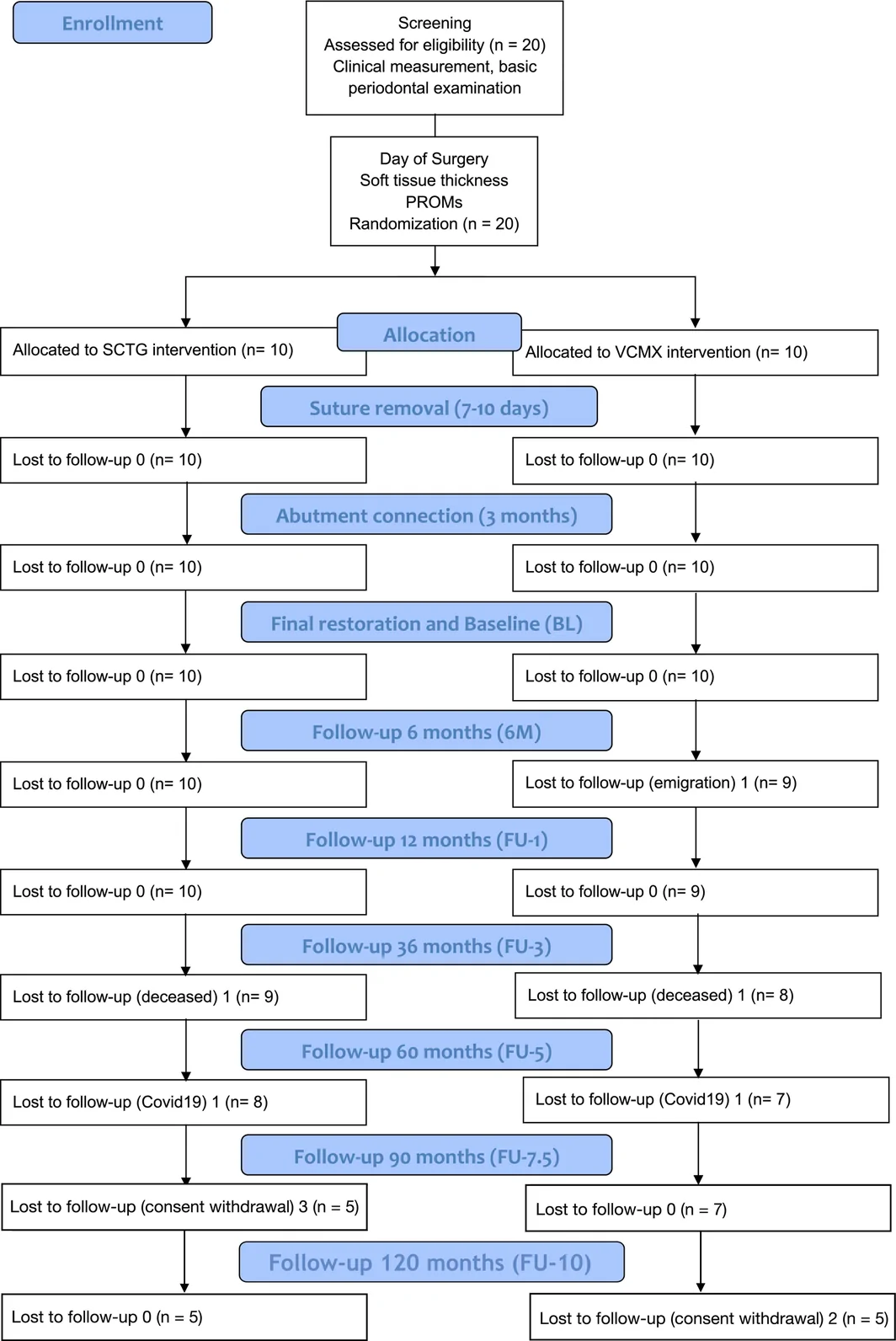
Consort flowchart.

**FIGURE 3:**
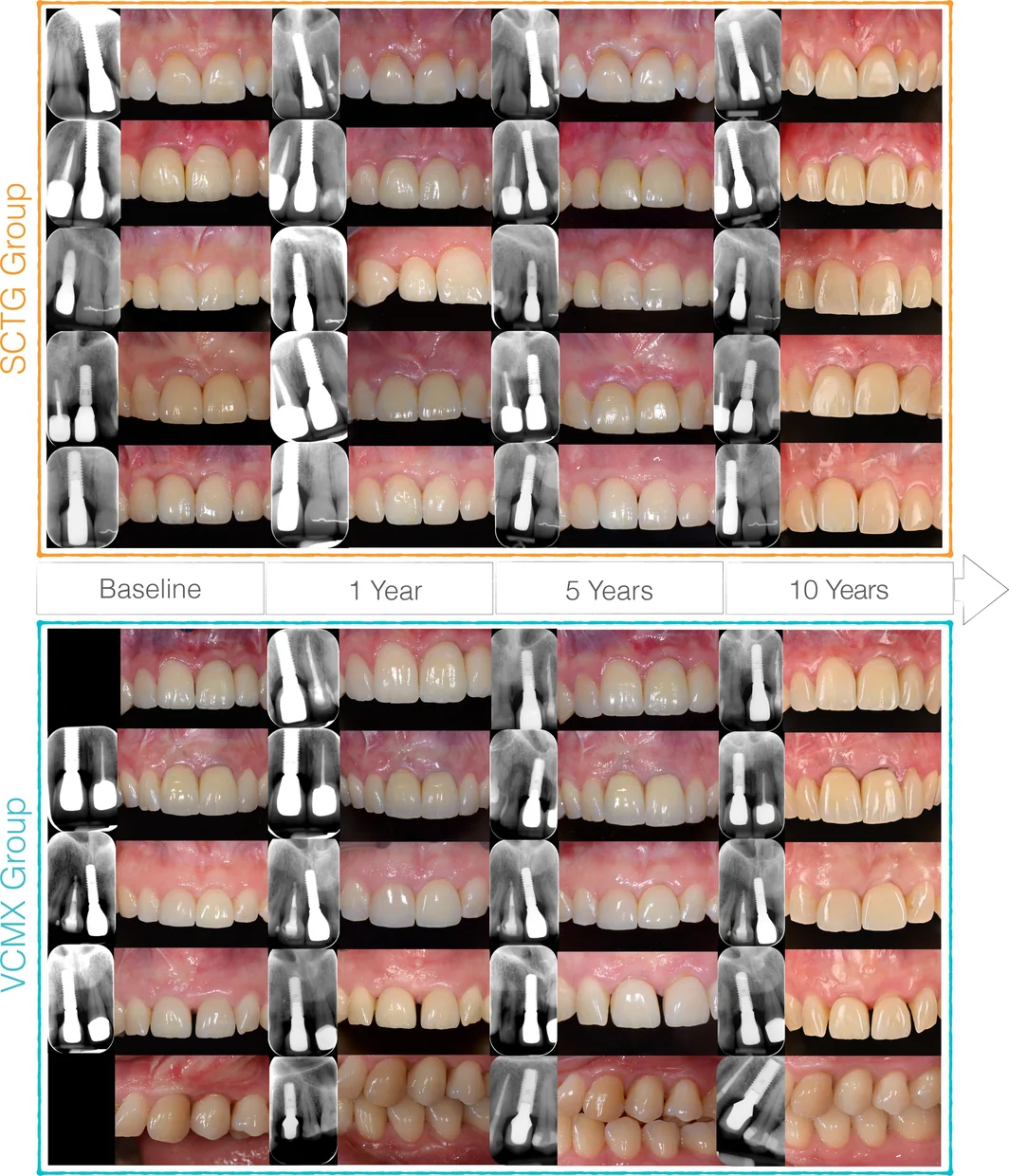
All patients available in each treatment group from baseline (BL) to re‐examination at the 10‐year follow‐up.

Patient demographics are summarized in Table 1. A total of 20 patients were enrolled and underwent soft tissue augmentation with either a volume‐stable collagen matrix (VCMX; *n* = 10) or a SCTG (*n* = 10). At baseline, smoking exposure was low in both groups, as heavy smokers (> 10 cigarettes/day) were excluded. During follow‐up, several patients were lost due to emigration, death, COVID‐19‐related restrictions, or withdrawal of consent. At the 10‐year evaluation, 10 patients (VCMX, *n* = 5; SCTG, *n* = 5) remained available for analysis, as shown in the CONSORT flow diagram (Figure 2). All implants and restorations remained functional throughout the observation period, resulting in a 100% survival rate up to 10 years. All patients remaining available at 10 years follow‐up are presented in Figure 3.

**TABLE 1:** Patient demographics at baseline.

	Group SCTG	Group VCMX
Gender		
*n* (female)	6	7
*n* (male)	4	3
Age		
Years, mean (SD)	43.4 (18.7)	44.1 (12.8)
Cigarettes per day (SD)	1.0 (2.5)	0.0 (0.0)
PD (mm) mean (SD)	3.0 (0.3)	3.1 (0.4)
BOP (%)	0.1	0.1
PCR (%)	0.1	0.0
MBL (mm) mean (SD)	−0.2 (0.4)	0.2 (0.7)

Abbreviations: BOP, bleeding on probing; MBL, marginal bone level; PCR, plaque control record; PD, probing depth; SCTG, subepithelial connective tissue grafts; SD, standard deviation; VCMX, volume‐stable collagen matrix.

### 3.2 Mucosal Thickness (Primary Outcome)

**FIGURE 4:**
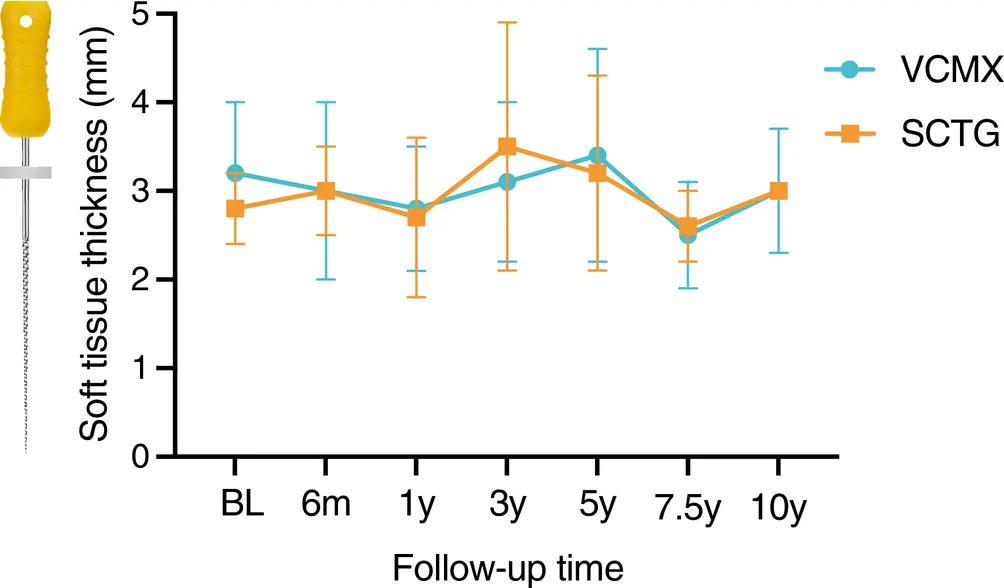
Line plots illustrating the mean soft‐tissue thickness over time for both treatment groups across all follow‐up time points. Error bars indicate SD.

Mucosal thickness values at all time points are presented in Table 2, and longitudinal changes are illustrated in Figure 4. At baseline, mucosal thickness was slightly greater in the VCMX group compared with the SCTG group (mean difference ≈ 0.7 mm [95% CI, −0.1 to 1.4]), although this difference was not statistically significant (*p* = 0.078). Over time, both groups showed a modest increase in tissue thickness. Throughout the study, differences between the two groups remained small (generally within ±0.2 mm) and were not statistically significant at any time point (*p* > 0.62). At the 10‐year evaluation, the adjusted between‐group difference in mucosal thickness was −0.02 mm (95% CI −0.99 to 0.96). As the lower bound of the CI remained above the prespecified non‐inferiority margin of −1 mm, non‐inferiority of VCMX compared with SCTG was shown. Linear mixed‐effects analysis showed no significant differences between groups over time and no interaction effect, indicating similar healing patterns for both treatments. Overall, both approaches showed a similar evolution, with no clinically meaningful differences in soft tissue thickness between the groups.

**TABLE 2:** Linear mixed‐effects models were used for mucosal thickness, probing depth (PD) and marginal bone level (MBL) and generalized estimating equation models were used for plaque control record and bleeding on probing (BOP).

Parameter	Timepoint	Group SCTG		Group VCMX		Comparison	
		Mean (SD)	Median (Q1; Q3)	Mean (SD)	Median (Q1; Q3)	Adjusted mean treatment difference (95% CI)	*p*
Mucosal thickness (mm)	Baseline	2.8 (0.4)	3.0 (2.5; 3.0)	3.2 (0.7)	3.0 (3.0; 3.9)	0.7 (−0.0; 1.4)	0.078
	6 months	3.0 (0.5)	3.0 (3.0; 3.2)	3.0 (1.0)	3.0 (2.0; 4.0)	−0.0 (−0.9; 0.8)	0.925
	1 year	2.7 (0.9)	2.5 (2.0; 3.8)	2.8 (0.7)	3.0 (2.0; 3.0)	−0.2 (−0.5; 1.0)	0.627
	3 years	3.1 (1.4)	3.0 (1.9; 4.2)	3.1 (0.9)	3.2 (2.6; 4.0)	0.1 (−0.7; 0.9)	0.807
	5 years	3.2 (1.1)	3.2 (3.0; 3.8)	3.4 (1.2)	3.0 (3.0; 4.0)	0.1 (−0.6; 0.9)	0.743
	7.5 years	2.6 (0.4)	2.7 (2.1; 3.0)	2.5 (0.6)	2.5 (2.0; 3.0)	−0.1 (−1.0; 0.7)	0.714
	10 years	3.0 (0.0)	3.0 (3.0; 3.0)	3.0 (0.7)	3.0 (3.0; 3.0)	−0.1 (−0.9; 0.9)	0.975
Probing depth (mm)	Baseline	3.0 (0.3)	3.0 (3.0; 3.2)	3.1 (0.4)	3.3 (3.2; 3.3)	−0.0 (−0.5; 0.4)	0.175
	6 months	2.9 (0.4)	2.8 (2.7; 3.2)	3.2 (0.3)	3.2 (3.2; 3.4)	0.3 (−0.1; 0.8)	0.300
	1 year	2.9 (0.7)	2.5 (2.5; 2.8)	2.6 (0.3)	2.7 (2.6; 2.7)	−0.3 (−0.8; 0.2)	0.642
	3 years	3.9 (0.5)	3.7 (3.7; 4.2)	3.4 (0.6)	3.7 (2.9; 3.7)	−0.5 (−1.1; −0.0)	0.035
	5 years	3.5 (0.6)	3.7 (3.3; 3.8)	3.3 (0.6)	3.3 (3.0; 3.6)	−0.1 (−0.7; 0.3)	0.977
	7.5 years	3.3 (0.5)	3.5 (3.0; 3.7)	3.2 (0.5)	3.0 (2.9; 3.5)	−0.1 (−0.6; 0.4)	0.926
	10 years	3.9 (0.7)	3.8 (3.5; 4.6)	3.1 (0.4)	3.1 (3.1; 3.3)	−0.7 (−1.3; −0.2)	0.005
Beeding on probing (%)	Baseline	0.1 (0.1)	0.0 (0.0; 0.2)	0.1 (0.2)	0.1 (0.0; 0.3)	0.0 (−0.1; 0.2)	0.118
	6 months	0.2 (0.2)	0.2 (0.0; 0.5)	0.2 (0.3)	0.0 (0.0; 0.4)	−0.0 (−0.3; 0.2)	0.426
	1 year	0.0 (0.0)	0.0 (0.0; 0.0)	0.2 (0.4)	0.0 (0.0; 0.2)	0.2 (−0.0; 0.4)	0.838
	3 years	0.2 (0.1)	0.3 (0.1; 0.3)	0.3 (0.3)	0.2 (0.0; 0.5)	−0.0 (−0.3; 0.3)	0.000
	5 years	0.0 (0.1)	0.0 (0.0; 0.0)	0.3 (0.2)	0.2 (0.2; 0.5)	0.2 (0.0; 0.5)	0.047
	7.5 years	0.3 (0.3)	0.3 (0.2; 0.7)	0.5 (0.3)	0.5 (0.3; 0.7)	0.0 (−0.2; 0.4)	0.438
	10 years	0.6 (0.2)	0.5 (0.5; 0.8)	0.3 (0.2)	0.3 (0.3; 0.6)	−0.2 (−0.5; 0.0)	0.150
Plaque control record (%)	Baseline	0.1 (0.0)	0.1 (0.0; 0.1)	0.0 (0.0)	0.0 (0.0; 0.0)	−0.0 (−0.1; 0.0)	0.180
	6 months	0.2 (0.2)	0.1 (0.0; 0.3)	0.1 (0.2)	0.0 (0.0; 0.2)	−0.0 (−0.3; 0.1)	0.568
	1 year	0.0 (0.0)	0.0 (0.0; 0.0)	0.1 (0.2)	0.0 (0.0; 0.0)	0.0 (−0.1; 0.2)	0.278
	3 years	0.0 (0.0)	0.0 (0.0; 0.1)	0.1 (0.3)	0.0 (0.0; 0.0)	0.0 (−0.2; 0.2)	0.037
	5 years	0.1 (0.1)	0.2 (0.2; 0.2)	0.2 (0.2)	0.3 (0.0; 0.3)	0.1 (−0.2; 0.3)	0.712
	7.5 years	0.3 (0.5)	0.1 (0.0; 0.1)	0.2 (0.4)	0.0 (0.0; 0.0)	−0.1 (−0.6; 0.4)	0.358
	10 years	0.1 (0.2)	0.0 (0; 0.3)	0.1 (0.2)	0.0 (0.0; 0.1)	0.0 (−0.3; 0.3)	1.000
Marginal bone level (mm)	Baseline	−0.2 (0.4)	−0.1 (−0.3; 0.2)	0.2 (0.7)	0.3 (0.1; 0.5)	0.4 (−0.2; 1.1)	0.164
	3 years	−0.5 (0.5)	−0.4 (−0.6; −0.3)	−0.5 (1.0)	−0.4 (−0.4; −0.0)	−0.0 (−0.7; 0.6)	0.972
	5 years	−0.6 (0.5)	−0.5 (−0.6; −0.3)	−0.4 (1.1)	−0.1 (−0.3; 0.0)	0.2 (−0.5; 0.9)	0.549
	7.5 years	−0.9 (1.1)	−0.3 (−1.3; −0.1)	−0.6 (1.0)	−0.4 (−0.5; −0.1)	0.1 (−0.5; 0.9)	0.705
	10 years	−1.2 (0.9)	−1.1 (−1.4; −0.4)	−0.5 (0.6)	−0.3 (−1.1; 0)	0.6 (−0.7; 1.4)	0.078

*Note:* Models compared treatment groups over time and were adjusted for treatment group, time and their interaction. Effect sizes are reported as adjusted mean differences with 95% CIs.

Abbreviations: CI, confidence interval; Q1, first quartile; Q3, third quartile; SCTG, subepithelial connective tissue grafts; SD, standard deviation; VCMX, volume‐stable collagen matrix.

### 3.3 Buccal Contour Changes of the Peri‐Implant Tissues

**FIGURE 5:**
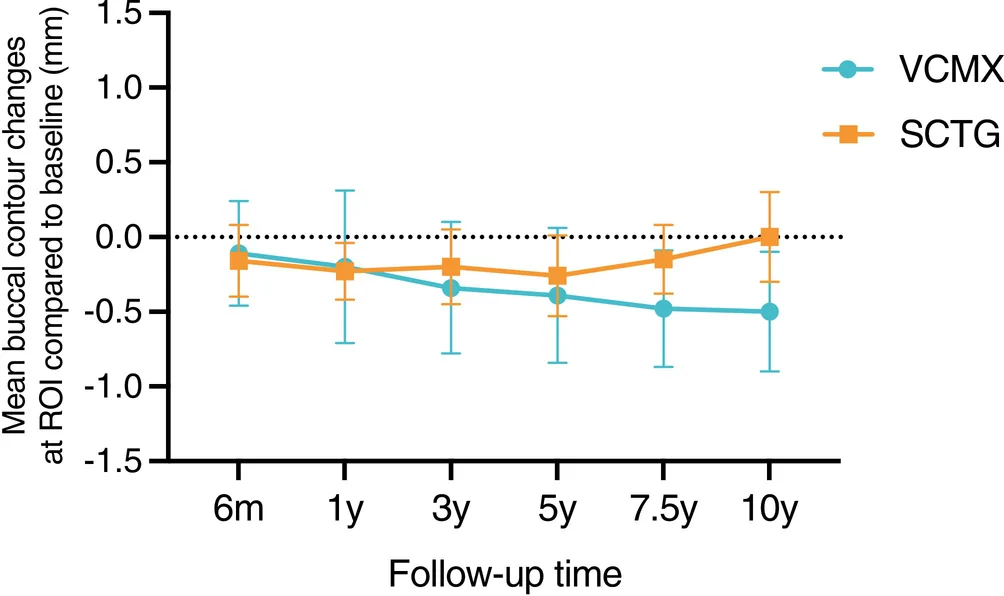
Line plots illustrating profilometric changes in buccal contour position relative to baseline within the region of interest over time for both treatment groups. Error bars indicate standard deviations (SD).

All contour changes across timepoints are reported in Table 3 and visually represented in Figure 5. Over time, no significant changes in volume were observed within the reference group at any follow‐up interval (*p* > 0.24). Intergroup differences remained small during the early and mid‐term follow‐up periods and were not statistically significant. However, at later time points, the VCMX group showed a tendency toward a greater contour decrease compared to group SCTG, with differences of approximately −0.29 mm at 7.5 years (95% CI, −0.60 to 0.02; *p* = 0.065) and −0.31 mm at 10 years (95% CI, −0.65 to 0.03; *p* = 0.074). Linear mixed‐effects analysis revealed a significant overall group‐by‐time interaction (*p* = 0.011), indicating that volumetric changes over time differed between the two treatment modalities.

**TABLE 3:** Profilometric contour changes within each treatment group compared with baseline across time points and between group differences over time.

Contour changes						
	Group SCTG		Group VCMX		Comparison	
Timepoint	Mean (SD)	Median (Q1, Q3)	Mean (SD)	Median (Q1, Q3)	Adjusted mean treatment difference (95% CI)	*p*
ΔBL—6 months	−0.1 (0.2)	−0.1 (−0.3; 0)	−0.1 (0.3)	0.0 (−0.2; 0.1)	0.0 (−0.2; 0.3)	0.746
ΔBL—1 year	−0.2 (0.1)	−0.2 (−0.3; −0.1)	−0.2 (0.5)	−0.1 (−0.3; 0.1)	0.0 (−0.2; 0.3)	0.843
ΔBL—3 years	−0.2 (0.2)	−0.1 (−0.3; 0.1)	−0.3 (0.4)	−0.2 (−0.7; −0.0)	−0.1 (−0.4; 0.1)	0.324
ΔBL—5 years	−0.2 (0.2)	−0.3 (−0.4; −0.1)	−0.3 (0.4)	−0.3 (−0.9; −0.1)	−0.0 (−0.3; 0.1)	0.469
ΔBL—7.5 years	−0.1 (0.2)	−0.2 (−0.3; −0.1)	−0.4 (0.4)	−0.3 (−0.9; −0.2)	−0.2 (−0.5; 0.0)	0.065
ΔBL—10 years	0.0 (0.3)	−0.1 (−0.1; 0.2)	−0.5 (0.4)	−0.4 (−0.9; −0.2)	−0.3 (−0.6; 0.0)	0.074

*Note:* Linear mixed‐effects models were used and adjusted for treatment group, time and their interaction. Effect sizes are reported as adjusted mean differences with 95% CIs.

Abbreviations: BL, Baseline; CI, confidence interval; Q1, first quartile; Q3, third quartile; SCTG, subepithelial connective tissue grafts; SD, standard deviation; VCMX, volume‐stable collagen matrix.

### 3.4 Clinical Outcomes

Clinical parameters are summarized in Table 2. Over time, PD increased significantly in the SCTG group at 3 and 10 years (Table 2). Between‐group differences were generally small, but statistically significant differences were observed at both 3 and 10 years (Table 2). At baseline, BOP was comparable between groups. During follow‐up, BOP was lower in the VCMX group at 3 years, with no differences at 10 years (Table 2). Plaque records (PCR) remained stable over time. A between‐group difference was observed at 3 years, with lower PCR values in the VCMX group, whereas no differences were detected at other timepoints, including 10 years. No cases of peri‐implantitis were identified in either group. At 10 years, the prevalence of peri‐implant mucositis was 80% in the VCMX group and 100% in the SCTG group, with no significant difference between groups (*p* = 1.000).

### 3.5 Radiographic Results

Radiographic outcomes (MBL) are presented in Table 2. Marginal bone levels changed significantly over time in both groups. At 10 years, mean marginal bone levels were −1.2 ± 0.9 mm in the SCTG group and −0.5 ± 0.6 mm in the VCMX group, with no significant difference between treatments (adjusted mean difference 0.6 mm [95% CI, −0.7 to 1.4]; *p* = 0.078) (Table 2). No significant between‐group differences were observed at any other timepoint.

### 3.6 Aesthetic Outcomes

All aesthetic outcomes are presented in Table 4. At baseline, mean PES values were comparable between groups, with no significant differences (adjusted mean difference of 0.8 [95% CI, −3.6 to 1.6]; *p* = 0.449). PES remained stable over time in both groups. A statistically significant difference was observed only at 6 months, with lower PES values in the VCMX group compared with the control group (Table 4). No other timepoints, including the 10‐year follow‐up, showed significant between‐group differences. No significant group‐by‐time interaction was detected (*p* = 0.599), indicating similar PES trajectories in both groups.

**TABLE 4:** Pink Aesthetic Scores (PES) in each treatment group across the different timepoints Linear mixed‐effects models were used and adjusted for treatment group, time and their interaction.

Aesthetic outcomes (PES)							
PES	Timepoint	Group SCTG		Group VCMX		Comparison	
		Mean (SD)	Median (Q1; Q3)	Mean (SD)	Median (Q1; Q3)	Adjusted mean treatment difference (95% CI)	*p*
	Crown delivery	8.6 (4.0)	10 (6; 11)	9.4 (1.1)	9 (9; 10)	0.8 (−2.0; 3.7)	0.449
	6 months	10.4 (3.0)	10 (10; 12)	8.7 (2.2)	8.5 (7.25; 9.75)	−1.8 (−4.8; 1.1)	0.034
	1 year	9.2 (2.4)	9 (8; 11)	8.9 (2.8)	8 (8; 11)	−0.3 (−3.2; 2.5)	0.363
	3 years	9.6 (2.5)	10 (9; 10)	9.0 (2.8)	9 (6.5; 11)	−0.6 (−3.5; 2.3)	0.363
	5 years	9.6 (3.0)	11 (8; 12)	10.1 (2.8)	10.5 (8.5; 12.5)	0.1 (−2.7; 3.1)	0.880
	7.5 years	9.2 (3.3)	11 (8; 11)	9.1 (3.2)	11 (7; 11)	−0.4 (−3.4; 2.4)	0.282
	10 years	10.6 (1.5)	11 (11; 11)	9.6 (3.6)	11 (8; 12)	−1 (−3.5; 1.5)	0.449

*Note:* Effect sizes are reported as adjusted mean differences with 95% CIs.

Abbreviations: CI, confidence interval; Q1, first quartile; Q3, third quartile; SCTG, subepithelial connective tissue grafts; SD, standard deviation; VCMX, volume‐stable collagen matrix.

### 3.7 Patient‐Reported Outcome Measures

All OHRQoL outcomes are presented in Table 5. At baseline, OHIP scores were comparable between groups, with no significant differences (*p* = 0.219) (Table 5). OHIP scores remained stable over time, with no significant changes within the groups. No significant differences between VCMX and the control group were detected at any timepoint. Furthermore, no significant group‐by‐time interaction was found (*p* = 0.246), indicating similar trajectories of oral health‐related quality of life over time in both groups.

**TABLE 5:** Oral Health Impact Profile‐14 (OHIP‐14) in each treatment group across the different timepoints.

Patient‐reported outcomes							
OHIP‐14	Timepoint	Group SCTG		Group VCMX		Comparison	
		Mean (SD)	Median (Q1; Q3)	Mean (SD)	Median (Q1; Q3)	Adjusted mean treatment difference (95% CI)	*p*
	Crown delivery	1.0 (1.7)	0.0 (0.0; 1.0)	2.4 (6.0)	0.0 (0.0; 0.5)	1.4 (−3.0; 5.9)	0.219
	6 months	0.8 (1.8)	0.0 (0.0; 0.0)	1.7 (3.6)	0.0 (0.0; 0.7)	0.7 (−2.2; 3.7)	0.570
	1 year	1.0 (2.2)	0.0 (0.0; 0.0)	1.1 (3.0)	0.0 (0.0; 0.0)	0.1 (−2.6; 2.9)	0.777
	3 years	0.0 (0.0)	0.0 (0.0; 0.0)	0.6 (1.1)	0.0 (0.0; 0.5)	0.5 (−0.2; 1.3)	0.705
	5 years	1.0 (1.4)	0.0 (0.0; 2.0)	0.8 (0.4)	0.0 (0.0; 0.0)	−0.9 (−2.2; 0.2)	0.850
	7.5 years	3.2 (7.2)	0.0 (0.0; 0.0)	0.7 (0.9)	0.0 (0.0; 0.7)	−2.4 (−8.3; 3.4)	0.200
	10 years	3.2 (7.1)	0.0 (0.0; 0.0)	0.8 (0.8)	1.0 (0.0; 1.0)	−2.4 (−6.5; 1.7)	0.256

*Note:* Generalized Estimating Equations (GEE) models were used and adjusted for treatment group, time and their interaction. Effect sizes are reported as adjusted mean differences with 95% CIs.

Abbreviations: CI, confidence interval; Q1, first quartile; Q3, third quartile; SCTG, subepithelial connective tissue grafts; SD, standard deviation; VCMX, volume‐stable collagen matrix.

## 4 Discussion

The present 10‐year follow‐up of a RCT assessed the long‐term performance of a volume‐stable collagen matrix (VCMX) compared to a SCTG for periimplant soft‐tissue volume augmentation at anterior implant sites. Although definitive conclusions cannot be drawn, given that only 10 of the 20 originally enrolled patients were available for re‐examination, the findings mainly showed:

— stable mucosal thickness over time, with no clinically meaningful differences between the groups;
— a tendency toward greater buccal contour reduction in the VCMX group at later time points;
— favorable aesthetic outcomes in both groups, without relevant differences;
— high levels of oral health–related quality of life, reflected by low OHIP‐14 scores.

### 4.1 Long‐Term Maintenance of Mucosal Thickness

The peri‐implant mucosal thickness seems to play a key role in maintaining soft tissue margin stability and marginal bone levels over time [3, 6, 33]. Therefore, soft tissue augmentation procedures have been increasingly advocated in cases of insufficient mucosal thickness to enhance tissue volume and improve buccal contour [34].

Autogenous SCTGs remain the gold standard for peri‐implant soft tissue augmentation, providing predictable increases in tissue thickness and keratinized tissue along with stable long‐term outcomes. However, their use is associated with increased morbidity related to graft harvesting, including postoperative pain and potential complications such as intraoperative bleeding, injury of the greater palatine artery, and tissue. For this reason, collagen matrices have been introduced as a less invasive alternative. Although several randomized clinical trials have compared these materials with SCTGs [14, 15, 23, 24, 29], the available evidence remains limited by short follow‐up periods. Without a long‐term follow‐up, the persistence of observed effects remains uncertain, limiting the strength of clinical recommendations. This is clinically relevant, as previous systematic reviews with short‐term data have suggested a tendency for greater shrinkage of soft‐tissue substitutes compared to SCTG over time [24, 27, 29]. A recent consensus report showed that differences in mucosal thickness gain become more pronounced with longer follow‐up, consistently favoring SCTG when treatment efficacy is prioritized. The weighted mean difference increased from 0.37 mm in studies with < 1 year of follow‐up to 0.79 mm in those with ≥ 1 year [35].

These differences favoring SCTG treatments were not confirmed in the present study, most likely owing to the limited sample size (*N* = 10) from the 20 patients originally recruited. It should also be noted that, at the time of study design (2011), no clinical data were available to inform a sample size calculation. Consequently, this series of studies contributed to sample size estimation in subsequent larger randomized clinical trials [14, 24]. Mucosal thickness, assessed by transmucosal probing, remained stable over time in the SCTG group, increasing slightly from approximately 2.7 mm at baseline to 3.0 mm at 10 years. In the VCMX group, a slight decrease was observed, from approximately 3.2 to 3.0 mm at 10 years. Although comparable 10‐year data are not available, these findings are consistent with those of a recent multicenter RCT with a 3‐year follow‐up, which reported a difference of approximately 0.2 mm in mucosal thickness between VCMX and SCTG [15], as well as with a recent noninferiority RCT reporting a similar difference (≈0.2 mm) [36].

### 4.2 Digital Volumetric Analysis and Contour Stability

Digital technologies are increasingly used in peri‐implant soft tissue assessment, allowing more precise and reproducible measurements [37]. In the present study, digital volumetric analysis complemented conventional clinical measurements, enabling a more comprehensive evaluation of soft tissue changes. No significant volumetric changes were observed within groups during early and mid‐term follow‐up and intergroup differences remained small. However, at later time points, the VCMX group showed a tendency toward greater buccal contour reduction, with a mean difference of −0.31 mm at 10 years. This greater shrinkage may be related to differences in tissue density and reduced vascularization of VCMX compared with SCTG [38]. Given the lack of comparable long‐term data, direct comparisons are limited. Nevertheless, a 3‐year RCT reported similar volumetric changes between materials, with most volume loss occurring during early healing and only minor changes thereafter [27]. At 3 years, buccal soft tissues were on average 0.35 mm thicker in the SCTG group, although the overall gain remained modest. It should also be considered that collagen matrices are typically thicker at placement than CTG, which may introduce bias when interpreting volumetric changes [27]. Overall, these findings suggest a potential tendency toward greater late contour reduction with VCMX. However, these small differences do not appear to compromise aesthetic outcomes and are likely imperceptible to patients, as supported by a recent systematic review and meta‐analysis comparing SCTG with soft tissue substitutes [26]. Importantly, the best treatment is not necessarily the one shown to be most efficacious in randomized clinical trials, but rather the one that aligns with the patient's individual values, preferences and expectations [39, 40].

### 4.3 Clinical Parameters

Clinical parameters, including PD, BOP, plaque levels, and marginal bone levels, showed no clinically meaningful differences between groups. However, the prevalence of peri‐implant mucositis was high in both groups (> 80%). Similar prevalences have been reported in other regions of Switzerland when applying the same case definition recommended by the EFP S3 guideline [31]. For example, a recent cross‐sectional study has reported peri‐implant mucositis in approximately 72% of patients using the same diagnostic criteria [41]. However, this high prevalence should be interpreted with caution, as it may partly reflect methodological aspects of outcome assessment rather than a true increase in disease burden. BOP is highly sensitive to variations in measurement and may be recorded using different criteria (e.g., punctiform vs. profuse bleeding). It is also influenced by probing technique, including the force applied [42]. In the present study, probing force was not standardized using a force‐calibrated probe (e.g., 0.2–0.25 N) which may have contributed to higher recorded BOP values. This is not unexpected, as probing force and BOP assessments are not consistently standardized across studies and may lead to overestimation of disease prevalence. BOP is not exclusively indicative of inflammation and may also be observed in clinically healthy peri‐implant tissues. Small variations in probing force can substantially affect BOP rates, for example, increasing probing force from 0.15 to 0.25 N significantly increases the frequency of BOP [43]. Taken together, reliance on dichotomous BOP outcomes may limit diagnostic accuracy and may partly explain the high prevalence of mucositis observed in this cohort.

At 10 years, mean marginal bone levels showed an adjusted mean difference of 0.6 mm in favor of VCMX; however, this was driven by a single outlier in the SCTG group. Given the small sample size, this outlier substantially influenced the estimate. After sensitivity analysis using mean substitution and repeating the mixed‐effects model, the difference was attenuated and no longer relevant (adjusted mean difference, 0.33 mm).

### 4.4 Aesthetic and Patient‐Reported Outcomes

Soft tissue phenotype modification may influence the aesthetic outcomes of implant‐supported restorations [7]. Implants receiving soft tissue augmentation tend to show more stable soft tissue margins over time, whereas non‐augmented sites are more prone to apical migration in the medium and long term [44]. In the present study, both grafting approaches resulted in excellent integration and stable aesthetic outcomes. Mean PES values remained stable over 10 years in both groups (SCTG: 10.6 ± 2.8 to 10.6 ± 1.5; VCMX: 9.6 ± 1.3 to 9.6 ± 3.6), with no statistically significant differences. These findings are consistent with previous trials reporting comparable aesthetic outcomes between VCMX and SCTG [36]. Although systematic reviews suggest that SCTG may provide superior aesthetic outcomes when the primary goal is to increase tissue thickness and volume, patient‐reported aesthetic satisfaction appears comparable between approaches [22, 26]. Therefore, treatment selection should incorporate patient preferences and tolerance for surgical morbidity. While SCTG may be preferred in highly demanding aesthetic cases, collagen matrices represent a suitable alternative for patients prioritizing reduced invasiveness.

### 4.5 Strengths and Limitations

A major strength of this study lies in the extended follow‐up period, which is rare in soft‐tissue augmentation trials in the aesthetic zone. However, the present trial has several limitations. First, the small sample size and high dropout rate may have introduced bias and further reduced the statistical power of the long‐term follow‐up. This limited the ability to detect statistically significant between‐group differences and may partly explain both the observed non‐inferiority of VCMX versus SCTG for mucosal thickness and the absence of significant differences in secondary outcomes. In addition, minor anatomical changes over time, such as soft‐tissue recession, may have affected measurement accuracy, since the ROI was defined at baseline and may not have fully corresponded to the same tissue area at follow‐up, potentially underestimating true volume loss. Future studies should evaluate coronal, middle, and apical zones separately using stable reference points. Furthermore, the indirect workflow used for STL acquisition may have introduced inaccuracies in volumetric analyses. Pre‐augmentation mucosal thickness was not recorded because this study represents the long‐term follow‐up of a previous RCT; therefore, baseline measurements reflected tissue conditions at crown delivery rather than the original gingival phenotype. Finally, probing force was not standardized using a force‐calibrated probe, which may have contributed to higher recorded BOP values and slight differences in PD between groups. Larger studies are needed to validate the present preliminary long‐term results.

## 5 Conclusions

Although definitive conclusions cannot be drawn, these preliminary long‐term findings showed no clinically relevant differences between SCTG and VCMX in terms of clinical, profilometric, and PROs.

## Author Contributions

All authors made substantial contributions to this study. D.S.T. and R.E.J. contributed to the conception and design of the study. F.J.S., M.G.L., N.N., T.J.W.G. contributed to the clinical phases of the study and collected the data. F.J.S. interpreted the data and performed the statistical analysis. F.J.S. and M.G.L. drafted the manuscript and R.E.J. and D.S.T. critically reviewed and revised it.

## Funding

This work was supported by Osteology Foundation, 1505680; Geistlich Pharma.

## Conflicts of Interest

The authors declare no conflicts of interest.

## Supporting information


**Table S1:** CONSORT checklist.

## Data Availability

The data that support the findings of this study are available on request from the corresponding author. The data are not publicly available due to privacy or ethical restrictions.

## References

[1] J. Cosyn , H. De Bruyn , and R. Cleymaet , “Soft Tissue Preservation and Pink Aesthetics Around Single Immediate Implant Restorations: A 1‐Year Prospective Study,” Clinical Implant Dentistry and Related Research 15, no. 6 (2013): 847–857. 22376232 10.1111/j.1708-8208.2012.00448.x

[2] G. M. Raghoebar , H. J. A. Meijer , B. van Minnen , and A. Vissink , “Immediate Reconstruction of Failed Implants in the Esthetic Zone Using a Flapless Technique and Autogenous Composite Tuberosity Graft,” Journal of Oral and Maxillofacial Surgery: Official Journal of the American Association of Oral and Maxillofacial Surgeons 76, no. 3 (2018): 528–533. 28972882 10.1016/j.joms.2017.09.005

[3] D. S. Thoma , N. Naenni , E. Figuero , et al., “Effects of Soft Tissue Augmentation Procedures on Peri‐Implant Health or Disease: A Systematic Review and Meta‐Analysis,” Clinical Oral Implants Research 29, no. Suppl 15 (2018): 32–49. 29498129 10.1111/clr.13114

[4] D. S. Thoma , J. Cosyn , S. Fickl , et al., “Soft Tissue Management at Implants: Summary and Consensus Statements of Group 2. The 6th EAO Consensus Conference 2021,” Clinical Oral Implants Research 32, no. Suppl 21 (2021): 174–180. 34145925 10.1111/clr.13798 PMC8596754

[5] D. Schneider , U. Grunder , A. Ender , C. H. F. Hämmerle , and R. E. Jung , “Volume Gain and Stability of Peri‐Implant Tissue Following Bone and Soft Tissue Augmentation: 1‐Year Results From a Prospective Cohort Study,” Clinical Oral Implants Research 22, no. 1 (2011): 28–37. 21039891 10.1111/j.1600-0501.2010.01987.x

[6] L. Tavelli , S. Barootchi , G. Avila‐Ortiz , I. A. Urban , W. V. Giannobile , and H.‐L. Wang , “Peri‐Implant Soft Tissue Phenotype Modification and Its Impact on Peri‐Implant Health: A Systematic Review and Network Meta‐Analysis,” Journal of Periodontology 92, no. 1 (2021): 21–44. 32710810 10.1002/JPER.19-0716

[7] S. P. Bienz , M. Pirc , S. N. Papageorgiou , R. E. Jung , and D. S. Thoma , “The Influence of Thin as Compared to Thick Peri‐Implant Soft Tissues on Aesthetic Outcomes: A Systematic Review and Meta‐Analysis,” Clinical Oral Implants Research 33, no. Suppl 23 (2022): 56–71. 35763024 10.1111/clr.13789 PMC9543651

[8] R. E. Jung , I. Sailer , C. H. F. Hämmerle , T. Attin , and P. Schmidlin , “In Vitro Color Changes of Soft Tissues Caused by Restorative Materials,” International Journal of Periodontics & Restorative Dentistry 27, no. 3 (2007): 251–257. 17694948

[9] G. M. Raghoebar , A. Korfage , H. J. A. Meijer , B. Gareb , A. Vissink , and K. Delli , “Linear and Profilometric Changes of the Mucosa Following Soft Tissue Augmentation in the Zone of Aesthetic Priority: A Systematic Review and Meta‐Analysis,” Clinical Oral Implants Research 32, no. Suppl 21 (2021): 138–156. 34642988 10.1111/clr.13759

[10] F. Cairo , L. Barbato , F. Selvaggi , M. G. Baielli , A. Piattelli , and L. Chambrone , “Surgical Procedures for Soft Tissue Augmentation at Implant Sites. A Systematic Review and Meta‐Analysis of Randomized Controlled Trials,” Clinical Implant Dentistry and Related Research 21, no. 6 (2019): 1262–1270. 31729830 10.1111/cid.12861

[11] T. De Bruyckere , J. Cosyn , F. Younes , et al., “A Randomized Controlled Study Comparing Guided Bone Regeneration With Connective Tissue Graft to Re‐Establish Buccal Convexity: One‐Year Aesthetic and Patient‐Reported Outcomes,” Clinical Oral Implants Research 31, no. 6 (2020): 507–516. 32011032 10.1111/clr.13587

[12] G. Zucchelli , L. Tavelli , M. K. McGuire , et al., “Autogenous Soft Tissue Grafting for Periodontal and Peri‐Implant Plastic Surgical Reconstruction,” Journal of Periodontology 91, no. 1 (2020): 9–16. 31461778 10.1002/JPER.19-0350

[13] E. G. Zuiderveld , H. J. A. Meijer , L. den Hartog , A. Vissink , and G. M. Raghoebar , “Effect of Connective Tissue Grafting on Peri‐Implant Tissue in Single Immediate Implant Sites: A RCT,” Journal of Clinical Periodontology 45, no. 2 (2018): 253–264. 28941303 10.1111/jcpe.12820

[14] C. H. F. Hämmerle , K. Jepsen , I. Sailer , et al., “Efficacy of a Collagen Matrix for Soft Tissue Augmentation After Implant Placement Compared to Connective Tissue Grafts: A Multicenter, Noninferiority, Randomized Controlled Trial,” Clinical Oral Implants Research 34, no. 9 (2023): 999–1013. 37403575 10.1111/clr.14127

[15] K. Jepsen , S. Jepsen , C. H. F. Hämmerle , et al., “Changes in Peri‐Implant Soft Tissue Dimensions Following Soft Tissue Contour Augmentation: A 3‐Year Follow‐Up of a Multi‐Center RCT,” Clinical Oral Implants Research 37, no. 3 (2026): 345–356. 41389213 10.1111/clr.70078

[16] K. M. Soileau and R. B. Brannon , “A Histologic Evaluation of Various Stages of Palatal Healing Following Subepithelial Connective Tissue Grafting Procedures: A Comparison of Eight Cases,” Journal of Periodontology 77, no. 7 (2006): 1267–1273. 16805692 10.1902/jop.2006.050129

[17] M. Stefanini , L. Tavelli , S. Barootchi , M. Sangiorgi , and G. Zucchelli , “Patient‐Reported Outcome Measures Following Soft‐Tissue Grafting at Implant Sites: A Systematic Review,” Clinical Oral Implants Research 32, no. Suppl 21 (2021): 157–173. 34642984 10.1111/clr.13767

[18] A. N. Zuercher , F. J. Strauss , R. Smirani , R. E. Jung , R. Gruber , and D. S. Thoma , “Clinicians' Stress Levels During Soft Tissue Augmentation Using Autogenous Connective Tissue Grafts: A Pilot Study,” Clinical Implant Dentistry and Related Research 28, no. 2 (2026): e70130. 41744355 10.1111/cid.70130 PMC12937924

[19] G. Brunello , G. H. Lin , I. Kopp , et al., “1st Global Consensus for Clinical Guidelines: Identifying a Core Outcome Set for Implant Dentistry in Edentulous Maxilla Rehabilitation,” Clinical Oral Implants Research 37, no. Suppl 30 (2026): S108–S120. 41732050 10.1111/clr.70075 PMC12930137

[20] H. De Bruyn , S. Raes , C. Matthys , and J. Cosyn , “The Current Use of Patient‐Centered/Reported Outcomes in Implant Dentistry: A Systematic Review,” Clinical Oral Implants Research 26, no. Suppl 11 (2015): 45–56. 26385620 10.1111/clr.12634

[21] D. S. Thoma , S. Sadilina , R. E. Jung , et al., “Patient‐ and Clinician‐Reported Outcomes and the Outcome Measures in Studies Reporting on Rehabilitation of the Edentulous Maxilla With Implant‐Supported Fixed Prosthesis Using Short, Standard‐Length and/or Zygomatic Implants: A Systematic Review of Clinical Studies Published in the Last 10 Years,” Clinical Oral Implants Research 37, no. Suppl 30 (2026): S247–S272. 41732055 10.1111/clr.14468 PMC12930138

[22] D. S. Thoma , F. J. Strauss , L. Mancini , T. J. W. Gasser , and R. E. Jung , “Minimal Invasiveness in Soft Tissue Augmentation at Dental Implants: A Systematic Review and Meta‐Analysis of Patient‐Reported Outcome Measures,” Periodontology 2000 91, no. 1 (2023): 182–198. 35950734 10.1111/prd.12465

[23] F. Cairo , C. Rupe , R. Cavalcanti , et al., “Cross‐Linked Volume‐Stable Collagen Matrix Versus Connective Tissue Graft for Soft Tissue Augmentation at Implant Site. A Non‐Inferiority, Multicenter Randomized Clinical Trial,” Clinical Oral Implants Research 37, no. 1 (2026): 45–56. 40984016 10.1111/clr.70050 PMC12767552

[24] J. Cosyn , C. Eeckhout , V. Christiaens , et al., “A Multi‐Centre Randomized Controlled Trial Comparing Connective Tissue Graft With Collagen Matrix to Increase Soft Tissue Thickness at the Buccal Aspect of Single Implants: 3‐Month Results,” Journal of Clinical Periodontology 48, no. 12 (2021): 1502–1515. 34605057 10.1111/jcpe.13560

[25] D. S. Thoma , M. Zeltner , M. Hilbe , C. H. F. Hämmerle , J. Hüsler , and R. E. Jung , “Randomized Controlled Clinical Study Evaluating Effectiveness and Safety of a Volume‐Stable Collagen Matrix Compared to Autogenous Connective Tissue Grafts for Soft Tissue Augmentation at Implant Sites,” Journal of Clinical Periodontology 43, no. 10 (2016): 874–885. 27310522 10.1111/jcpe.12588

[26] A. Ramanauskaite , S. Sadilina , F. Schwarz , E. A. Cafferata , F. J. Strauss , and D. S. Thoma , “Soft‐Tissue Volume Augmentation During Early, Delayed, and Late Dental Implant Therapy: A Systematic Review and Meta‐Analysis on Professionally Determined Esthetics and Self‐Reported Patient Satisfaction on Esthetics,” Periodontology 2000, ahead of print, April 16, 2025, 10.1111/prd.12628. 40241249

[27] L. Surdiacourt , V. Christiaens , T. De Bruyckere , et al., “A Multi‐Centre Randomized Controlled Trial Comparing Connective Tissue Graft With Collagen Matrix to Increase Soft Tissue Thickness at the Buccal Aspect of Single Implants: 3‐Year Results,” Journal of Clinical Periodontology 52, no. 1 (2025): 92–101. 38485651 10.1111/jcpe.13975

[28] M. G. Liguori , F. J. Strauss , T. J. W. Gasser , R. E. Jung , N. Naenni , and D. S. Thoma , “Soft Tissue Augmentation at Single Implants With Collagen Matrix or Connective Tissue Graft: 7.5‐Year Follow‐Up of a RCT,” Clinical Implant Dentistry and Related Research 28, no. 2 (2026): e70146. 41978333 10.1111/cid.70146 PMC13077265

[29] J. Cosyn , C. Eeckhout , T. De Bruyckere , et al., “A Multi‐Centre Randomized Controlled Trial Comparing Connective Tissue Graft With Collagen Matrix to Increase Soft Tissue Thickness at the Buccal Aspect of Single Implants: 1‐Year Results,” Journal of Clinical Periodontology 49, no. 9 (2022): 911–921. 35781692 10.1111/jcpe.13691

[30] T. Berglundh , G. Armitage , M. G. Araujo , et al., “Peri‐Implant Diseases and Conditions: Consensus Report of Workgroup 4 of the 2017 World Workshop on the Classification of Periodontal and Peri‐Implant Diseases and Conditions,” Journal of Clinical Periodontology 45, no. Suppl 20 (2018): S286–S291. 29926491 10.1111/jcpe.12957

[31] D. Herrera , T. Berglundh , F. Schwarz , et al., “Prevention and Treatment of Peri‐Implant Diseases‐The EFP S3 Level Clinical Practice Guideline,” Journal of Clinical Periodontology 50, no. Suppl 26 (2023): 4–76. 10.1111/jcpe.13823 37271498

[32] R. Fürhauser , D. Florescu , T. Benesch , R. Haas , G. Mailath , and G. Watzek , “Evaluation of Soft Tissue Around Single‐Tooth Implant Crowns: The Pink Esthetic Score,” Clinical Oral Implants Research 16, no. 6 (2005): 639–644. 16307569 10.1111/j.1600-0501.2005.01193.x

[33] A. Puisys and T. Linkevicius , “The Influence of Mucosal Tissue Thickening on Crestal Bone Stability Around Bone‐Level Implants. A Prospective Controlled Clinical Trial,” Clinical Oral Implants Research 26, no. 2 (2015): 123–129. 10.1111/clr.12301 24313250

[34] L. Tavelli , D. Thoma , M. E. Galarraga‐Vinueza , M. Romandini , and S. Barootchi , “Soft Tissue Substitutes: Current Biomaterials and Indications at Teeth and Implant Sites,” Journal of Periodontal Research, ahead of print, December 31, 2025, 10.1111/jre.70066. 41474175

[35] R. E. Jung , K. Becker , S. P. Bienz , et al., “Effect of Peri‐Implant Mucosal Thickness on Esthetic Outcomes and the Efficacy of Soft Tissue Augmentation Procedures: Consensus Report of Group 2 of the SEPA/DGI/OF Workshop,” Clinical Oral Implants Research 33, no. Suppl 23 (2022): 100–108. 35763020 10.1111/clr.13955 PMC9543632

[36] D. S. Clem , P. K. McClain , M. K. McGuire , et al., “Harvest Graft Substitute for Soft Tissue Volume Augmentation Around Existing Implants: A Randomized, Controlled and Blinded Multicenter Trial,” Journal of Periodontology 95, no. 8 (2024): 740–748. 38071454 10.1002/JPER.23-0305

[37] F. J. Strauss , A. Gil , R. Smirani , A. Rodriguez , R. Jung , and D. Thoma , “The Use of Digital Technologies in Peri‐Implant Soft Tissue Augmentation—A Narrative Review on Planning, Measurements, Monitoring and Aesthetics,” Clinical Oral Implants Research 35, no. 8 (2024): 922–938. 38308466 10.1111/clr.14238

[38] R. Smirani , F. J. Strauss , S. Hitz , R. E. Jung , R. Kraus , and D. S. Thoma , “Tissue Integration and Vascularization of Volume Collagen Matrices and Connective Tissue Grafts Following Peri‐Implant Soft Tissue Augmentation: A Secondary Histological Analysis,” Swiss Dental Journal 135, no. 2 (2025): 37–42. 40329895 10.61872/sdj-2025-02-04

[39] A. Chow , S. Purkayastha , D. Dosanjh , R. Sarvanandan , I. Ahmed , and P. Paraskeva , “Patient Reported Outcomes and Their Importance in the Development of Novel Surgical Techniques,” Surgical Innovation 19, no. 3 (2012): 327–334. 22158844 10.1177/1553350611426011

[40] D. S. Thoma and F. J. Strauss , “On the Discrepancy Between Professionally Assessed and Patient‐Reported Outcome Measures,” Journal of Periodontal & Implant Science 52, no. 2 (2022): 89–90. 35505571 10.5051/jpis.225202edi01 PMC9064775

[41] E. Couso‐Queiruga , M. Fonseca , V. Chappuis , et al., “Bone‐Level Versus Tissue‐Level Titanium Dental Implants: A Comparative Cross‐Sectional Study,” Journal of Clinical Periodontology 53, no. 4 (2026): 529–538. 41431257 10.1111/jcpe.70080 PMC12972610

[42] A. Monje and G. E. Salvi , “Diagnostic Methods/Parameters to Monitor Peri‐Implant Conditions,” Periodontology 2000 95, no. 1 (2024): 20–39. 38923148 10.1111/prd.12584

[43] J. A. Gerber , W. C. Tan , T. E. Balmer , G. E. Salvi , and N. P. Lang , “Bleeding on Probing and Pocket Probing Depth in Relation to Probing Pressure and Mucosal Health Around Oral Implants,” Clinical Oral Implants Research 20, no. 1 (2009): 75–78. 19126110 10.1111/j.1600-0501.2008.01601.x

[44] M. Stefanini , S. Barootchi , M. Sangiorgi , et al., “Do Soft Tissue Augmentation Techniques Provide Stable and Favorable Peri‐Implant Conditions in the Medium and Long Term? A Systematic Review,” Clinical Oral Implants Research 34, no. Suppl 26 (2023): 28–42. 37750532 10.1111/clr.14150

